# Empathy and Emotional Intelligence in Adolescent Cyberaggressors and Cybervictims

**DOI:** 10.3390/ijerph17134681

**Published:** 2020-06-29

**Authors:** Lucía Segura, Jesús F. Estévez, Estefanía Estévez

**Affiliations:** Department of Health Psychology, Universidad Miguel Hernández de Elche, 03202 Elche, Alicante, Spain; lucia.segura01@goumh.umh.es (L.S.); jesus.estevez01@goumh.umh.es (J.F.E.)

**Keywords:** emotional intelligence, empathy, cybervictimization, cyberaggression, adolescence

## Abstract

The main objective of the present research was to examine the role played by emotional intelligence in its three dimensions—emotional attention, emotional clarity, and emotion regulation—and by empathy in its four dimensions—perspective-taking, empathic understanding, empathic stress, and empathic joy—in cyber violence, both in aggressors and victims. A total sample of 1318 adolescents (47% boys; aged between 11 and 17 years), enrolled in four secondary compulsory education schools in Spain, participated in the study. The results indicated that, regarding emotional intelligence, cyberaggressors showed statistically significant differences in the dimension of emotion regulation. Participation in violent online behaviors is associated with a lower capacity to regulate emotions; cybervictims showed statistically significant differences in the three dimensions of emotional intelligence. Regarding empathy, cyberaggressors obtained statistically significant group differences in three of these dimensions: perspective-taking, empathetic joy, and empathic stress. Finally, the empathy dimensions for the cybervictimization groups did not show significant mean differences, indicating that there was no statistical relationship between the degree of cybervictimization and the individual’s empathy. These findings stress the relevance of emotion regulation in cyberviolence in students in adolescence and allow us to understand the different roles it plays for offenders and victims.

## 1. Introduction 

School violence is a very relevant social problem at the international level, affecting a growing number of children and adolescents [1,2,3]. This social problem implies significant negative consequences for the physical, emotional, and psychological well-being of the individuals involved [4,5]. Bullying in the educational context includes any unjustified behavior at the physical, verbal, psychological, or relational level, which implies an aggressive behavior repeated over time towards one or more peers with the intention of harming them [6].

Therefore, the conventional definition of bullying includes three features: the aggressor’s intentionality, the repetition over time, and the unequal power between aggressor and victim [7]. The aggressor is aware of their attitude towards the victim and their intention is to dominate and control the other person [8], creating an environment of violence where the victim cannot easily defend themselves [9,10].

In recent years, in addition to this traditional form of peer bullying, abuse and intimidation through information and communication technologies (ICTs) have increased among adolescents [11,12]. This form of abuse, known as cyberbullying, also involves unwarranted and intentional attacks carried out repeatedly against victims who cannot easily defend themselves but, in this case, it is exercised through the use of new technologies and electronic devices, such as computers or mobile phones [13,14]. Recent research indicates the growing existence of cyberbullying situations, probably due to the huge expansion of new devices, such as smartphones and tablets, whose daily availability is increasing among the younger population [15,16].

Despite the similarity to bullying, characteristics such as the anonymity of the aggressors or the larger audience for humiliation provide cyberbullying with its own identity and, in fact, it seems to imply even more negative and devastating consequences for victims than direct aggression [17], with bullying and cyberbullying being closely related to each other, and turning direct violent behavior into the predictor of cyberggression with the greatest explanatory weight [18].

Studies that have investigated the prevalence and effects of cyberbullying have arrived at different conclusions. Modecki, Minchin, Harbaugh, Guerra, and Runions [19] concluded from their meta-analyses that the prevalence values of this problem ranged from 4 to 36% for cybervictimization and between 16 and 18% for cyberaggression. In a cross-sectional study involving seven European countries (Germany, Greece, Iceland, the Netherlands, Poland, Romania, and Spain), it was found that the highest rate of cyber victimization was in Romania (37.3%) and the lowest in Spain (13.3%) [20]. Another study on cyberbullying also found variations in the prevalence rates of self-reported victimization, with rates of 55% in North America and Asia, 25% in Canada, and 30% in Europe [21]. Some of the variations in percentages among these studies could be explained by the different conceptualizations of the problem, as well as by the variety of instruments and methodologies used in the research. In the report published in 2016 by the Save The Children Foundation [22], based on a survey of 21,487 secondary compulsory education (SCE) students aged 12 to 16, the authors indicate that 6.9% of the children claim to have been a “cybervictim” and 3.3% “cyberaggressors”. From this perspective, it is clear that there is an increase in cyberbullying, but few works have jointly analyzed the relationships between cyberbullying and the variables of psychological adjustment [23].

Interest in studying emotional intelligence (EI) in the adolescent population has increased, particularly in recent decades, due to the evidence shown by some studies on its relevant role in cyberbullying [24,25,26]. The scientific literature on EI has indicated that the way people tend to process emotional information during stressful situations is a key aspect of general well-being, healthy functioning, and quality social relationships [27]. Various investigations have shown that students with high levels of EI can regulate and manage their own emotions and those of others to better restore their emotional adjustment and general well-being [28,29,30]. 

One of the most solid theoretical approaches with an empirical basis in EI research is the approach proposed in Mayer and Salovey’s model of EI [31]. This model distinguishes four abilities in the construct of EI: perception, facilitation, understanding, and the regulation of emotions. In particular, *emotional perception* refers to the capacity to be aware of one’s own and others’ emotions; *emotional facilitation* consists of the ability to use emotions to communicate feelings; *emotional understanding* is the capacity to comprehend how emotions can change and combine over time and to appreciate their meanings; lastly, *emotion regulation* consists of the ability to be open to feelings and to manage emotions.

Recent studies of cyberaggressors show that they generally obtain low scores in social and emotional skills [32]. In particular, in works such as the one carried out by Fernández-González, Calvete, Orue, and Echezarraga [33], the authors conclude that adolescents with limited EI competencies show fewer resources to resolve interpersonal conflicts and make more use of aggression as a means of problem-solving, to the detriment of more adaptive strategies. In fact, Garaigordobil [34] found an association between participating in cyberbullying and showing lower levels in all three dimensions of EI: attention, clarity, and emotion regulation. Adolescents with a greater variety of affective abilities based on the understanding and management of their own emotions may channel their anger more constructively, and not resort to violence as a means to overcome their frustration when facing life events in adolescence. [34].

Previous research on cyberbullying and cybervictimization has indicated that students with high levels of EI exhibit more positive social behaviors and are less victimized by their peers [25,35,36]. Additionally, the development of emotional skills that are typical of EI (paying attention to, understanding, and knowing how to manage emotions) can reduce the risk in adolescent cybervictims of eventually developing psychological problems as a consequence of cyberbullying [26]. EI seems to play a moderating role between being a victim in a cyberbullying situation and the emotional impact of this event on the victim [25,37]. 

Deficits in EI or its dimension have been positively related to cybervictimization [24], as recently indicated by Elipe et al. [25], who found that, when taking into account the dimensions of EI, cybervictims have greater competence in attending to their emotions, but lower competence in understanding and regulating them [25,38,39]. These results suggest the important role that the emotion regulation dimension plays in cybervictims. Some authors have stressed that deficits in the ability to express and regulate emotions can act as a risk factor, predicting both cybervictimization and victimization [40,41]. In this sense, it seems that the coping style that victims tend to use to defend themselves is confrontation focused on emotion, which implies that they present greater emotional attention and, given the complex situation they undergo in the educational context, they also have more difficulties in properly managing their emotions [40,42]. Other authors, like Erath, Flanagan, and Bierman [43], have pointed out that cybervictims often present more difficulties in adjusting their daily life emotions, a fact that can sometimes eventually develop into a greater inability to adopt the others’ perspectives, which negatively affects the victims’ empathic responses and capacities [44].

The empathic ability is a construct strongly related to EI, as empathy is one of the skills closely associated with the understanding and use of emotions. Ciarrochi, Chan, and Caputi [45] observed in their study that adolescents who presented high EI also tended to show high empathy levels. In fact, the studies focused on empathy have consistantly concluded that the lack of this ability is involved in the quality of interpersonal relationships, because it hinders harmonious interactions with others [46]. On the contrary, in several works, empathy deficits have been associated with the individual’s difficulty to establish positive social interactions, an increase of interpersonal conflicts, and even the development of behavioral problems during adolescence [47]. *Empathy* can be defined as the ability to understand the emotional state of others and to internalize that emotional state [3]. Thus, empathy implies an emotional response that begins with the comprehension of the other’s emotions, which produces similar emotions to those of the other [48].

Empathy is a multidimensional construct in which cognitive and affective–emotional dimensions converge, such as: a) *perspective-taking,* which refers to the intellectual ability to put oneself in the place of another person; b) *empathic comprehension,* the capacity to recognize and understand other people’s intentions, moods, and impressions; c) *empathetic stress,* the ability to share someone else’s negative emotions; and d) *empathetic joy,* the capacity to share another person’s positive emotions [49,50,51].

In the meta-analysis carried out by Zych, Farrington, and Ttofi [52], the authors conclude that empathy is one of the most relevant constructs involving social interactions established in situations of bullying and cyberbullying. In a deeper analysis, studies examining empathy from a multidimensional perspective in cases of cyberbullying have shown different results. Concerning cyberaggressors, Renati, Berrone, and Zanetti, for example [53], found lower scores in affective empathy in this group, whereas in the study carried out by Rodríguez-Hidalgo, Solera, and Calmaestra [54], cyberaggressors presented lower levels of affective and cognitive empathic skills. The nature of cyberspace facilitates aggressors’ low affective and cognitive empathy, as they have difficulty recognizing the effect of their behavior on the victim, making it hard for them to suffer remorse or social rejection for their behavior [55,56]. The fact that cyberbullying takes place through technological supports facilitates the disinhibition of the behaviors of the aggressors, who act impulsively, without thinking about the consequences of their actions. When the aggressions take place in cyberspace, the physical distance between the victim and the aggressor prevents the perception of the damage and, thus, of the bully’s empathic response [57]. Because of the invisibility between the bully and victim in aggressive relationships, there is little opportunity for empathy or remorse for the victim’s feelings or for the extent to which this violence can affect them [58].

Few studies have investigated the association between empathy and cybervictimization, and moreover, they reached opposing conclusions. In this sense, the results from this research have indicated that victims have normally high levels of empathy, which is related to a greater sensitivity to the intentions of the aggressor. [59,60]. In contrast, Schultze-Krumbholz and Scheithauer [61] found lower empathy scores in cybervictims compared to uninvolved students. 

### The Present Study

In short, peer bullying through new technologies is a recent and growing problem, and previous works, many of which are based on bullying, have confirmed the important role that psychological variables play in its comprehension, development, and maintenance. Even though several precedent studies have shown the relation between EI and empathy, both in cybervictims and cyberaggressors, some questions remain unanswered in the current scientific literature. In the first place, does such a relationship exist, and in what sense, if EI is considered from a multidimensional perspective (analyzing the skills of attention, clarity, and the regulation of emotions)? Secondly, what is the association between cyberaggressors and cybervictims like? Finally, are the emotional and cognitive dimensions of empathy different in cybervictims and cyberaggressors?

Based on these research questions, this study was organized around two main objectives: (1) to analyze EI both in cyberaggressors and cybervictims, considering the three dimensions of emotional attention, clarity, and emotion regulation; and (2) to analyze empathy in cyberaggressors and cybervictims, taking into account both its cognitive and affective dimensions.

A different emotional profile in cyberaggressors and cybervictims was expected, concerning the way they perceive, understand, and regulate their emotions and those of others. The main contribution of the present study was to analyze the different dimensions or skills integrated into the constructs of empathy and EI, and to compare them in cyberaggressors and cybervictims, and with those of adolescents who are uninvolved in cyberbullying situations.

## 2. Materials and Methods 

### 2.1. Participants

Analyses from the present study are based on data from a representative sample of Spanish secondary school students who were recruited through random cluster sampling in the geographical areas of the Valencian Community, Aragon, and Andalusia. The primary sampling units were the the urban and rural geographic areas of the three communities. The secondary units were the public and private secondary schools in each area. Classrooms were not considered as tertiary units, as all classrooms from first to fourth grade of the selected schools were included in the study. A series of prior analyses of differences of means was conducted on the target variables of the study as a function of the location of the school and of its public or private condition, without finding any statistically significant differences. The total sample was composed of 1318 adolescents (47% boys), whose ages ranged from 11 to 17 years (Mean = 13.8, *SD* = 1.32). Students were equally distributed by academic level: 24.7% in first grade, 27.3% in second grade, 23.7% in third grade, and 24.3% in the fourth grade of SCE.

### 2.2. Instruments

Cyberaggression (CYB-AGRES; Buelga and Pons [62])**.** This scale attempts to establish the number of cyberaggressions committed during the past year using a mobile phone. This instrument is composed of 10 items that assess behaviors involving aggression, such as: (1) Harassment (e.g., “I have insulted or ridiculed people with messages or calls”); (2) Persecution (e.g., “I have threatened people to frighten them”); (3) Vilification (e.g., “I have told lies or false rumors about someone”); (4) Violation of Privacy (e.g., “I have told other people’s secrets to annoy them”); (5) Social Exclusion (e.g., “I made calls and did not speak, or I told people to be online and did not meet them”); (6) Identity Theft (e.g., “I have pretended to be someone else to say or do bad things by mobile phone or internet”). This scale uses a four-point Likert scale ranging from 1 (*never*) to 4 (*always*) for responses. The global scale showed a Cronbach alpha of 0.94 in the present sample; for the two dimensions, the values obtained were 0.93 and 0.86, respectively.

Cybervictimization (CYBVIC-R; Buelga, Cava, and Musitu [63]). This scale consists of 24 items that measure the harassment suffered through mobile phones and the internet during the last year: Harassment (e.g., “I have been insulted or ridiculed by someone on social networks or in groups like WhatsApp to really hurt me”), Persecution (e.g., “They have forced me through threats to do things I did not want to do on the internet or a mobile”), Denigration (e.g., “They have told my secrets or revealed personal things about me without my permission on social networks or in groups”), Violation of Intimacy (e.g., “I have been recorded or they have taken humiliating photos of me without my permission and they have distributed them on social networks”), Social Exclusion (e.g., “I have been ignored and they have not answered my messages or things that I have sent to groups or social networks to make me feel bad”), Identity Theft (e.g., “They have used my profile or my accounts without me being able to prevent it”). Responses were registered on a five-point scale ranging from 1 (*never*) to 5 (*very often*). The scale also has three items about the time and frequency of the aggressions and the perpetrator. The Cronbach alpha obtained for this scale was 0.95.

Trait Meta-Mood Scale (TMMS; Salovey, Mayer, Goldman, Turvey, and Palfai [64], in the Spanish version adapted by Fernández-Berrocal, Extremera, and Ramos [65]). This scale consists of 22 items that measure, on a five-point Likert scale (1 = *strongly disagree*, 5 = *strongly agree*), the three dimensions of EI: emotional attention (e.g., “I pay close attention to how I feel”), emotional clarity (e.g., “I am clear about my feelings”), and emotion regulation (e.g., “When I’m angry, I try to change my mood”). Cronbach’s alpha for this scale in the present sample was 0.91, and for the three dimensions, it was 0.91, 0.86, and 0.87, respectively.

Cognitive and Affective Empathy Scale (TECA; López-Pérez et al. [49]). This instrument is composed of 33 items that measure the cognitive and affective dimensions of empathy through a five-point Likert scale (1 = *never*, 5 = *always*). The Cognitive Empathy dimension includes the subdimensions of Perspective-Taking (e.g., “I try to understand my friends, looking at situations from their perspective”) and Emotional Comprehension (e.g., “I know when someone tries to hide their true feelings”). The Affective Empathy dimension includes the subdimensions of Empathetic Stress (e.g., “Unless it is something very serious, it is hard for me to cry about what happens to others”) and Empathetic Joy (e.g., “When something good happens to someone else, I feel happy”). In the present sample, the value obtained for the Cronbach’s alpha was 0.83 for the global scale; all subscales had an alpha that exceeded 0.75, except for Empathic Stress, which reached 0.60.

### 2.3. Procedure

This study is part of a broader work on problem behavior in the adolescent stage in the Spanish population, with the ethics committee aproval of the Universidad Miguel Hernández de Elche for the research project with the reference PSI2015-65683-P. In the first place, a letter describing the research was sent to the schools selected to participate in the study. Secondly, we contacted the headmasters who had showed their willingness to collaborate in the investigation, and organized a briefing with the teaching and educating staff to inform them of the purposes of the study. Once participation was agreed upon, a letter describing the study was sent to the parents, requesting their passive consent for their children to participate in the research. The administration of the instruments was carried out after the students had also provided their written consent, and during their regular classes. Participants anonymously and voluntarily filled out the instruments in their respective classrooms. The order of the administration of the instruments was counterbalanced in each classroom and school. The surveys that were suspicious in terms of their response patterns were not coded in the database (these surveys represented 1% of the total original samples).

### 2.4. Data Analysis

The study sample was classified into three groups, according to their degree of participation in cyberaggression or cybervictimization situations. This degree of participation was estimated using the 25th and 75th percentiles in adolescent scores on the cyberaggression and cybervictimization scales. The resulting categories correspond to Low, Medium, and High cyberaggression, and Low, Medium, and High cybervictimization. The number of participants in each group were: Low Cybervictimization: 421; Medium Cybervictimization: 594; High Cybervictimization: 303; Low Cyberagression: 659; Medium Cyberagression: 378; High Cyberagression: 281.

To test the hypotheses, analysis of variance (ANOVA) was performed. The independent variables were those of classification, which allowed for comparing the results by group. The dependent variables were EI (Table 1 and Table 2) and the four subdimensions of Cognitive and Affective Empathy (Table 3 and Table 4). Additionally, a post hoc test was performed. In all cases, the Levene test [66] indicated the existence of the heterogeneity of variances in all the variables, considering both the groups formed by the degree of cyberaggression and by cybervictimization. Based on the results of that test, the Games–Howell post hoc test was applied to determine between which specific groups there were significant statistical differences, because this is an appropriate method for multiple comparisons without making assumptions about the homogeneity of variances [67,68].

We also calculated the effect size through Cohen’s *d* statistic, whose value is indicative of large differences in means when its absolute value is greater than 0.80, moderate differences when it is between 0.50 and 0.80, and small differences when it is between 0.20 and 0.50 [69]. The value was estimated considering the differences of means between the groups of High and Low Cyberaggression (Table 1 and Table 3) on the one hand, and High and Low Cybervictimization on the other (Table 2 and Table 4). All calculations were carried out with the statistical program IBM SPSS 23 (IBM, New York, NY, USA).

## 3. Results

All tables show the means, standard deviations, and the results of the ANOVA. Table 1 shows the relationship between EI and the degree of involvement of adolescents in cyberaggression. The results indicate statistically significant differences in the Emotion Regulation dimension (*F* = 5.11, *p* < 0.01). It was observed that participation in violent online behaviors (groups with medium and high violence) was associated with a lower capacity to regulate emotions. The post hoc test indicated a specific significant difference between the participants with Low and High Cyberaggression. However, no statistically significant differences were found between the groups in the dimensions of Emotional Attention or Emotional Clarity.

Table 2 shows the results of EI, this time considering the Low, Medium, and High Cybervictimization groups. The results revealed statistically significant differences in the three dimensions of EI. Cybervictims had the highest Emotional Attention (*F* = 5.05, *p* < 0.01). Specifically, the Games–Howell test indicated that the difference between the means of the High and Low Cybervictimization groups was significant, with means of 3.37 and 3.16, respectively.

Contrary to what occurred with the dimension of Attention, the other two dimensions of EI showed decreasing values as the degree of cybervictimization increased. Thus, highly victimized adolescents obtained the lowest scores on Emotional Clarity (*F* = 7.16, *p* < 0.001). The Games–Howell test indicated that there was no significant difference of means between the Low and Medium Cybervictimization groups, but there was a significant difference between these two groups and the High Cybervictimization group.

Regarding Emotion Regulation (*F* = 13.80, *p* < 0.001), the Games–Howell test indicated significant differences among the three groups, that is, the High Cybervictimization group had a significantly lower mean score compared both to the Medium and the Low Cybervictimization groups. In turn, the Medium Cybervictimization group had a significantly lower mean than the Low Cybervictimization group, which showed the greatest capacity for emotion regulation.

Table 3 shows the results of the ANOVA in the four subdimensions of empathy for the Low, Medium, and High Cyberaggression groups. The results revealed statistically significant group differences in three of these subdimensions. In the Perspective-Taking dimension, there was a significant global difference of means (*F* = 6.93, *p* < 0.01), by which the Games–Howell test specifically discriminated between the High and Low Cyberaggression groups. The difference showed that an adolescent with a high tendency toward cyberaggression had a lower capacity to take the other’s perspective. Along the same lines, the Games–Howell test indicated that there was a significant difference in the empathetic joy subdimension between the High Cyberaggression group and the other two groups, which were not different from each other. 

In the case of empathetic stress, there was a significant global difference in means (*F* = 3.87, *p* < 0.05), where the Low Cyberaggression group had a significantly lower mean than the other two groups. Finally, the degree of cyberaggression did not seem to be significantly associated with the emotional understanding subdimension (*F* = 2.43, *p* = 0.09), although, in any case, this subdimension obtained the lowest value in the High Cyberaggression group.

The results of the ANOVA of the subdimensions of empathy for the groups of Low, Medium, and High Victimization are shown in Table 4. Apparently, there were no significant differences between the means, indicating that there was no statistical relationship between the degree of cybervictimization and the empathy of a subject when considered globally.

## 4. Discussion

This research had a primary objective to analyze empathy and EI in cybervictims and cyberaggressors. We examined the three dimensions of EI proposed by Salovey and Mayer [70], and also the different dimensions of empathy (i.e., perspective-taking, empathetic understanding, empathic stress, and empathetic joy). In general, the results of this study regarding cyberaggressors indicated the existence of a relationship between low empathy and low emotion regulation with aggressive behavior against peers, with this finding coinciding with the results of previous research [71,72,73].

Particularly, when examining EI from a multidimensional perspective, our analyses showed that cyberaggressors presented greater difficulty in the emotion regulation component; that is, their ability to manage emotions appropriately. On the contrary, no difficulties were found in aggressors in the dimensions of attention and emotional clarity. This result indicates that cyberaggressors may attend to and understand their emotions to the same extent as an adolescent not involved in a bullying situation, but that their main emotional deficit may be linked to their ability to regulate feelings, emotions, and behaviors in a socially acceptable way. Thus, they tend to choose expressions of anger and frustration that they cannot control, instead of using positive strategies in interpersonal situations [35]. In this line, previous studies [33] conclude that adolescents with limited EI skills have fewer resources to resolve interpersonal conflicts and resort more to aggression as a means of solving problems, to the detriment of more adaptive strategies. 

Our results suggest that adolescents who attack through technological means can attend to and understand their emotions normally. This result is along the lines of some works that state that, in cyberaggression, we must interpret the other person’s psychological state because they are not present, whereas, in the case of bullying, their physical presence places us directly in front of the victim, with the consequences of emotional impact that this entails [74]. Thus, the type of emotional processing underlying cyberbullying may be more elaborate, more leisurely, and less spontaneous, versus the faster and more direct processing in the case of bullying. These differences could lead the person to assess their abilities to attend to and regulate their emotions differently [74,75].

On the other hand, our data seem to confirm that cybervictims have deficits in all the dimensions of EI. Victimized adolescents presented less emotional clarity, as well as a lower ability to comprehend and manage their own emotions. These findings are in line with those presented by Ortega et al. [41], who highlighted that victimized students showed a lower ability to regulate their emotions. Research usually points to a deterioration in cybervictims’ emotional skills, as they tend to have more difficulty in recognizing and expressing their emotions appropriately and managing their own and others’ emotions, especially when these emotions are more intense and, at the same time, their regulatory capacity is diminished [76]. On the other hand, it has been observed that an adequate development of EI skills (paying attention to emotions, understanding them, and knowing how to manage them) can be a protective factor, reducing the risk in adolescent cybervictims of eventually developing psychological problems as a consequence of cyberbullying [32]. However, the existing scientific literature about EI in cybervictims is negligible despite the importance of this construct.

Empathy, in its two dimensions—affective and cognitive—seems to also be negatively affected in cyberaggressors, given the findings from the present study. Different studies confirm that adolescents and children who are habitual observers of violence in mass media, such as television, the internet, or video games, tend to behave more aggressively and have less empathy with the victims of assault [77,78,79,80]. When people frequently observe violent behaviors, both directly and on screen, it is more probable for them to normalize violence as a way to resolve problems and to behave in life. They become insensitive to others’ pain, considering aggressive behavior to be appropriate and normative in conflict resolution, associating violence with the domination of others and the relatively easy gaining and achievement of certain objectives [34,81,82]. Other studies, such as that of Hinduja and Patchin [83], conclude that cyberaggressors also show greater deficits in empathy, such as the violence they exercise through new technologies, which prevent them from observing the direct consequences of their behavior in the victim. Cyberaggressors’ cognitive and emotional empathy may also be affected by the repeated violence they exercise towards their victims, perhaps as a consequence of a desensitization process or moral disengagement, as some authors have indicated [84].

Finally, concerning the empathic ability of the victims, our results indicated that there are no statistically significant differences in any dimension of this construct. A meta-analysis on empathy and cyberbullying among schoolchildren conducted by Zych, Baldry, Farrington, and Llorent [85] discovered that cybervictims obtained the same score as non-cybervictims in the empathy construct. These results are consistent with empirical evidence showing that cybervictims have no lack of empathy [56]. Despite the relevance of the study of empathy in cybervictimization, the existing scientific literature in this regard is very limited, so the results of this study represent a significant advance in our context.

### Limitations and Future Research

The authors acknowledge some limitations in the present study, which should be taken into account for research in the future. The first limitation is related to the fact that the work is based on data of a cross-sectional nature, which makes it impossible to affirm the existence of causal relations among the variables analyzed. Second, we must keep in mind that, although the instruments were administered anonymously, self-administered questionnaires could show response bias, which may affect the generalizability and validity of the findings. In future research, it is recommended to incorporate data from other informants, such as teachers and parents, whose responses could lead to a broader approach to the problem studied, adding a complementary view to that of students. Third, these results are circumscribed to the adolescent stage (aged from 11 to 17), which means they cannot be generalized to students of other educational levels or ages (early childhood education, primary education, and higher education), or school contexts from other cultures. However, we again highlight the contribution of the present work in terms of better knowing the role of EI and empathy in the understanding of cyberaggressors’ and cybervictims’ emotions and the ways they manage them. This is important due to the scarcity of studies focused on this population and topic, and still less from this multidimensional perspective. We believe that it is essential to implement training and educational programs that involve the direct engagement and cooperative work of families, students, and teachers for the responsible use of new technologies. 

In terms of future research, it would be interesting to hear the views of a greater number of young people and to find out if these views are similar or different from the adult vision of politics in the future.

## 5. Conclusions

The findings of the present work contribute to our better understanding of the important role played by empathy and EI in situations of cybervictimization and cyberbullying. Conclusions from the present study entail important contributions to the intervention and prevention of this social problem, which affects many adolescents. An important goal that would be interesting to work on in the educational context to reduce situations of bullying is empathic skills training. Emotional awareness is necessary to prevent disruptive behavior as a whole. Thus, according to our results, interventions should focus on addressing empathic skills in their two dimensions, as well as on emotion regulation skills to reduce the activation caused by anger or frustration in cyberaggressors [86,87].

We know from the empirical evidence that the importance of emotion regulation is such that deficits in EI can negatively affect school performance and other important emotional indicators, such as stress, self-esteem, or depression [35]. In this sense, studies have demonstrated that adolescents obtaining high scores in EI normally have a more positive perception of their social relationships and groups of friends [88,89]. Therefore, promoting the learning and training of the emotional and empathic skills in the principal socialization contexts—such as the school and the family—may give students a key opportunity to develop adequate strategies for social interactions [90,91,92]. In parallel, educational practices aimed at teachers should be implemented so that they can obtain the necessary strategies and resources to intervene in cases of cyberbullying [93]. These practices should also be focused on developing students’ sensitivity so that they are aware of signs of bullying situations [34,87]. In fact, some intervention programs, such as the TEI [94], whose objective is improving school integration with the aim of preventing situations of bullying, based on emotional peer tutoring and the promotion of empathy, has shown evident effective results from a prevention perspective. 

In short, this study is a novel work that provides promising results on the EI and empathy of cyberaggressors, but above all, of cybervictims, as research focused on examining empathy from a multidimensional perspective is very scarce. The deepening of our understanding of cyberbullying can help to design more efficient intervention programs to prevent and reduce this problem that has a growing presence in our societies, which is of great educational, clinical, and social relevance.

## Figures and Tables

**Table 1:** Means, standard deviations (in parentheses), and results of the ANOVA of EI for the groups of Low, Medium, and High Cyberaggression.

EI Dimension	Low Cyber-Aggression	Medium Cyber-Aggression	High Cyber-Aggression	*F*	*p*	*d*
Attention	3.24 (0.98)	3.28 (0.91)	3.31 (0.82)	0.76	0.47	0.08
Clarity	3.38 (0.84)	3.32 (0.83)	3.31 (0.68)	1.24	0.29	−0.09
Regulation	3.47 (0.94) ^a^	3.37 (0.89) ^a,b^	3.30 (0.81) ^b^	5.11	<0.01	−0.19

**Note.** a,b: Different letters indicate that the mean difference is statistically significant at the 0.05 level in the Games–Howell test. EI: Emotional intelligence.

**Table 2:** Means, standard deviations (in parentheses), and results of the ANOVA of EI for the groups of Low, Medium, and High Cybervictimization.

EI Dimension	Low Cyber-Victimization	Medium Cyber-Victimization	High Cyber-Victimization	*F*	*p*	*d*
Attention	3.16 (0.97) ^a^	3.28 (0.86) ^a,b^	3.37 (0.92) ^b^	5.05	<0.01	0.22
Clarity	3.42 (0.85) ^a^	3.37 (0.72) ^a^	3.22 (0.80) ^b^	7.16	<0.01	−0.24
Regulation	3.53 (0.92) ^a^	3.41 (0.83) ^b^	3.20 (0.92) ^c^	13.80	<0.01	−0.36

**Note**. a,b,c: Different letters indicate that the mean difference is statistically significant at the 0.05 level in the Games–Howell test. EI: Emotional intelligence.

**Table 3:** Means, standard deviations (in parentheses), and results of the ANOVA of empathy for the groups of Low, Medium, and High Cyberaggression.

Empathy Subdimension	Low Cyber-Aggression	Medium Cyber-Aggression	High Cyber-Aggression	*F*	*p*	d
Perspective-taking	3.53 (0.90) ^a^	3.45 (0.77) ^a^	3.34 (0.73) ^b^	6.93	<0.01	−0.23
Emotional understanding	3.53 (0.82)	3.61 (0.66)	3.48 (0.64)	2.43	0.09	−0.08
Empathetic stress	2.37 (0.77) ^a^	2.49 (0.72) ^b^	2.48 (0.62) ^b^	3.87	<0.05	0.15
Empathetic joy	4.06 (0.86) ^a^	4.09 (0.71) ^a^	3.90 (0.81) ^b^	6.51	<0.01	−0.20

**Note.** a,b: Different letters indicate that the mean difference is statistically significant at the 0.05 level in the Games–Howell test. EI: Emotional intelligence.

**Table 4:** Means, standard deviations (in parentheses), and results of the ANOVA of empathy for the groups of Low, Medium, and High Cybervictimization.

Empathy Subdimension	Low Cyber-Victimization	Medium Cyber-Victimization	High Cyber-Victimization	*F*	*p*	*d*
Perspective-taking	3.47 (0.93)	3.47 (0.73)	3.43 (0.86)	0.51	0.60	−0.04
Emotional understanding	3.51 (0.83)	3.54 (0.64)	3.53 (0.76)	0.96	0.39	0.04
Empathetic stress	2.43 (0.78)	2.46 (0.66)	2.41 (0.73)	0.08	0.93	−0.04
Empathetic joy	4.05 (0.88)	4.02 (0.70)	3.92 (0.90)	2.24	0.11	−0.10
